# Integrating community health assistant-driven sexual and reproductive health services in the community health system in Nyimba district in Zambia: mapping key actors, points of integration, and conditions shaping the process

**DOI:** 10.1186/s12978-019-0788-4

**Published:** 2019-08-13

**Authors:** Joseph M. Zulu, John Kinsman, Anna-Karin Hurtig, Charles Michelo, Asha George, Helen Schneider

**Affiliations:** 1 Department of Public Health, School of Medicine, University of Zambia, School of Public Health, P.O. Box 50110, Lusaka, Zambia; 2 Department of Global Health and Epidemiology, Umeå University, 90185 Umeå, SE Sweden; 3 School of Public Health and SAMRC Health Services to Systems Unit, University of the Western Cape, Robert Sobukwe Road, Bellville, Cape Town, 7535 South Africa

**Keywords:** Community health system, Community health assistants, Sexual and reproductive health services

## Abstract

**Introduction:**

Although large scale public sector community health worker programs have been key in providing sexual and reproductive health (SRH) services in low- and middle-income countries, their integration process into community health systems is not well understood. This study aimed to identify the conditions and strategies through which Community Health Assistants (CHAs) gained entry and acceptability into community health systems to provide SRH services to youth in Zambia. The country’s CHA program was launched in 2010.

**Methodology:**

A phenomenological design was conducted in Nyimba district. All nine CHAs deployed in Nyimba district were interviewed in-depth on their experiences of navigating the introduction of SRH services for youth in community settings, and the data obtained analyzed thematically.

**Results:**

In delivering SRH services targeting youth, CHAs worked with a range of community actors, including other health workers, safe motherhood action groups, community health workers, neighborhood health committees, teachers, as well as political, traditional and religious leaders. CHAs delivered SRH education and services in health facilities, schools, police stations, home settings, and community spaces. They used their health facility service delivery role to gain trust and entry into the community, and they also worked to build relationships with other community level actors by holding regular joint meetings, and acting as brokers between the volunteer health workers and the Ministry of Health. CHAs used their existing social networks to deliver SRH services to adolescents. By embedding the provision of information about SRH into general life skills at community level, the topic’s sensitivity was reduced and its acceptability was enhanced. Further, support from community leaders towards CHA-driven services promoted the legitimacy of providing SRH for youth. Factors limiting the acceptability of CHA services included the taboo of discussing sexuality issues, a gender discriminatory environment, competition with other providers, and challenges in conducting household visits.

**Conclusion:**

Strengthening CHAs’ ability to negotiate and navigate and gain acceptability in the community health system as they deliver SRH, requires support from both the formal health system and community networks. Limitations to the acceptability of CHA-driven SRH services are a product of challenges both in the community and in the formal health system.

## Plain English summary

We conducted a qualitative study to identify the conditions and strategies through which community health assistants (CHAs) gain entry and acceptability in community health systems for promoting sexual and reproductive health (SRH) in Zambia. We collected data through face to face interviews with all the nine CHAs who were deployed in Nyimba district to understand the acceptability process.

The interviews showed that CHAs successfully delivered SRH education and services in health facilities, schools, police stations, home settings, and community spaces. The CHAs worked with a range of community actors, including health workers, safe motherhood action groups, community health workers, neighborhood health committees, teachers, as well as political, traditional and religious leaders in delivering SRH services. The CHAs collaborated with these community level actors by holding regular joint meetings, and by acting as brokers between the volunteer health workers and the Ministry of Health. The use of existing social networks to deliver SRH services to adolescents as well as support from community leaders also facilitated acceptability of CHA SRH services in the community. Barriers to delivering the SRH services include the taboo of discussing sexuality issues, gender inequalities, CHAs increasingly being drawn into facility based roles and lack of transport for conducting outreach activities.

In conclusion, there is need for actors from both the community and formal health system to provide support to CHAs for them to effectively deliver SRH services.

## Introduction

Many young people in Zambia and other low and middle income countries (LMICs) experience sexual and reproductive health (SRH) challenges such as teen pregnancy and marriage, unmet family planning needs, STIs and HIV/AIDS [1]. National community-based health worker programs have been key in providing SRH services in LMICs [2–6]. In the face of a significant human resources for health gap and to improve accessibility to SRH services, especially among young people, Zambia developed the National Community Health Assistant Strategy in 2010. The strategy proposed a new cadre called Community Heath Assistant (CHA) who would be integrated into the formal health system, with a base in health posts [7, 8]. The CHAs come from and are selected by the community they serve and they differ from other CHWs in a number of ways: compared to most CHWs, CHAs have a longer (one year) and standardised training which is provided by the Ministry of Health, they have a standardised monthly salary, and they are on the government payroll. Their activities include screening patients (taking vital signs), as well as testing and treating minor illnesses such as malaria, diarrhoea, respiratory tract infections and burns/sores. CHAs also deliver SRH services which include conducting testing, treatment of HIV and STIs as well as maternal and child health activities [9–11].

A growing body of international guidance on the scaling up and sustainable implementation of community-based health interventions recognises the contribution of both the formal health sector and community factors to the success of programs [7]. For programs such as that of the CHAs to succeed, they need to thus be integrated into formal health systems while being simultaneously embedded in and supported by communities [12–14]. Furthermore, community health worker (CHW) programs are complex interventions which need to be adapted to both the national or formal health system and also local community systems [6, 13, 14].

Community health systems have been defined as the space between the formal health system and the community, often existing in a “grey zone” between public, non-governmental and private health systems [15]. This grey zone consists of household-level caregivers, volunteers and informal health providers, organizational intermediaries such as non-governmental organizations, religious and sporting groups, as well as other government sectors such as housing, education, and social development [11]. However, community health systems remain insufficiently characterized [16, 17]. There is inadequate definition or characterisation of the local political structures, the approaches that shape the relationships surrounding CHWs, and of the community health system itself [18]. Some actors that CHWs work with in the community health system are also often invisible or not well characterised [17].

Integration into community health systems is influenced by the “plural set of providers, diverse norms and values, as well as less formal and horizontal mechanisms which shape coordination and accountability at community level” [19, 20]. With respect to sexual and reproductive health, prevailing gender norms are one of the key contextual factors that affect acceptability of community-based health worker interventions into health systems [21, 22]. For example, women may not be comfortable disclosing pregnancies to male CHWs or discussing SRH issues with CHWs of the opposite sex [21].

Integration is also significantly influenced by the professional identity of CHWs and how they position themselves, as community or health system players. In contrast to small scale community based programmes, the more formalised (i.e. trained and remunerated) CHWs of national programmes may see themselves more as health workers than as community members [17]. Resource constraints such as inadequate drugs also affect the quality of services delivered by community-based health workers and the acceptability of their services by the community [9, 10, 22, 23]. In Zambia, health systems factors which have affected the performance of CHAs include limited integration of CHAs into systems of district health governance, inadequate supervision, and relatively heavy involvement of CHAs in the health posts (as opposed to working out in the community) [10].

While these challenges have been acknowledged, the approaches, strategies or ways through which community-based health workers balance the mix of role identities between belonging to the community and the formal health system as they deliver SRH services, or the means by which they negotiate with the various elements of the community health system to gain acceptability, have not been fully explored [24]. Given the sensitivities involved with sexual and reproductive health, understanding this integration into community health systems is important. This study therefore sought to explore how CHAs integrate SRH into their relationships and interactions with the communities they serve. Specifically, the study aimed to identify the conditions and strategies through which community health assistants (CHAs) gained entry and acceptability into community health systems for SRH targeting youth in Zambia. It also mapped actors and the places that CHAs worked with in delivering SRH services. The paper mainly focuses on the family planning component of SRH services (e.g. condoms and contraception). We focused on these services because providing family planning services to adolescents is complex due to religious and cultural barriers [25].

### The community health assistant strategy and SRH in Zambia

In Zambia, the maternal mortality ratio is still high at 398/100,000 live births, with 30% of the mortality due to unsafe abortions, of which 80% take place in adolescents [26]. In addition, 30% of girls aged 15 to 19 years begin child bearing, 25% of married girls aged 15–19 have an unmet need for family planning, and HIV prevalence among youths aged 15–24 stands at 7% [26–28]. Early pregnancy and marriage are a public health problem because they are associated with school dropout. Low education, in turn, is a recognised risk factor for sexual risk taking, which ultimately can lead to unwanted pregnancies and increased risk of maternal mortality [29].

These SRH challenges have been worsened by a huge shortage in human resources for health. In the face of this shortage, roughly 23,500 CHWs in small scale programmes including community-based distributors (who provide family planning products in the community), have been active in providing outreach services during the past 10 years.

The CHWs work closely with CHAs when delivering SRH services. On average, two CHAs are deployed in each health post. By 2017, over 1700 CHAs had been deployed [30]. A health post usually has one trained staff, who is a nurse and support staff such as cashiers, cleaners and guards.

At the local level, the monitoring of CHA activities is conducted by CHA supervisors and by the Neighbourhood Health Committees (NHCs). The NHCs are community structures that help in managing and organising health programmes at community level. The members of the NHCs are elected by the community, and include community health workers [9]. The supervision process at the local level takes the form of checking the reports prepared by CHAs and also mentorship processes. Through community meetings at the local level, NHCs review the different issues that affect the work of CHAs.

#### The formal and community health systems in Zambia

The formal health system in Zambia consists of five levels of service delivery. The highest level is 3rd Level Hospitals, followed by 2nd Level Hospitals and 1st Level Referral Hospitals. Health centres (HCs) and health posts (HPs), which make up the highest proportion of health facilities in Zambia, are located at the lowest levels of service delivery, the community health system. CHAs, NHCs and other actors work with the health centres and health posts in delivering and monitoring health services (including SRH services) in the communities.

## Methodology

The methods section has been organised around the key features of the consolidated criteria for reporting qualitative research (COREQ) checklist. The key issues from the checklist that we have discussed within the methodology section include detailed information on the study setting, how participants were selected, previous knowledge or relationship between researcher and respondents, as well as the processes through which rigour was ensured during data collection and analysis.

### Study design

The study took a phenomenological approach, which focused on a single case, Nyimba district. The phenomenological approach was used as it is “the participants’ perceptions, feelings, and lived experiences that are paramount and that are the objective of study” [31]. This approach is well suited to understanding CHAs’ daily life experiences with regard to providing SRH services and the actors that they work with at community level, the places they work in, and also the factors that shape their integration into the community health system [9]. This work builds on the first author’s PhD work which analysed the factors that have shaped the integration process of national community health worker programs into health systems in LMICs [6].

### Study setting

Nyimba district is among the eight districts in Eastern Province of Zambia. Data were collected in all the health posts where CHAs were deployed. Situated along the Great East Road, the district is 350Km east of Lusaka, the Capital City of Zambia, and 250 Km from Chipata, the provincial headquarters of Eastern Province. Based on the 2010 census of population, the district was projected to have had a population of 97,296 in 2017. The average population growth rate between the periods 2000 to 2010 was estimated at 2% (2.1% for males and 1.9% for females). Nyimba district covers an area of 10,943 sq. Km and density of 9.217/km^2^. The district’s main economic activities comprise game management areas and subsistence farming [32].

With regard to health services, the District has a District Hospital, 12 rural health centres and 7 health posts. Physical accessibility to most of the health facilities in Nyimba District is through feeder roads which are often impassable in the rainy season, making it hard to reach the more remote health centres [32].

Despite some health successes, Nyimba district faces significant health and health care challenges, manifest by a high disease burden, inadequate professional medical staff (especially midwives), inadequate laboratory supplies and equipment, and low outreach activity [32]. The District straddles a major highway connecting Zambia to neighbouring countries, with truck and other stopping points contributing to a steady increase in HIV and STI prevalence [33]**.**

The health governance system at the community level has two parts. One part is the health post or facility system which is coordinated by the in-charge at the health facility, which reports to the District Health Office, and from the district level to the provincial and national levels. The other part is the community health sector which is coordinated by the neighborhood health committees, and the political governance system. The neighborhood health committees (NHC) participate in developing plans and budgets for the health facilities, and they also participate in selecting and supervising CHAs. The NHCs also coordinate the other actors such as the safe motherhood action groups and CHWs.

The political governance system is made up of the traditional leadership and elected political actors. The chief coordinates the traditional governance system and is closely assisted by village headmen. The councilor is the highest elected political leader at community level, who reports to the member of parliament at district level. The councilor is assisted by the ward development committee (WDC) chairperson in planning and delivering development projects such as sources of water. Nyimba, like other districts in Zambia, is predominantly a Christian district although other religions, such as Islam and Hinduism, are also practiced. Religious leaders comprise an important part of the community leadership system.

### Sample size

Nine CHAs who were deployed in Nyimba District in August 2012 were included in the study. This is the total number of CHAs deployed and working in the District at the time of the study. We identified the CHAs from the District’s human resources for health records.

### Data collection

In line with the phenomenological approach, we used in-depth interviews as the main method of data collection, allowing us to gain insight into the world of the CHAs, and their experiences in delivering SRH services, as well as their interactions with the actors with whom they work. Data were collected by the first author, who has extensive experience as well as postgraduate training in qualitative data collection, specifically with CHAs, as his PhD topic was on the Zambian CHA program. The author was able to capitalize on his existing knowledge and relationships to develop rapport with the CHAs, who readily volunteered their stories about the factors that have shaped their integration – or lack of integration – into the community health system. The interviews loosely followed an interview guide and lasted between 40 and 55 min. The key questions were centered on how the CHAs negotiate with the various actors in the community in ways that are acceptable as they deliver what are considered as culturally sensitive services.

### Data analysis

Interviews were recorded digitally and later transcribed verbatim. The interviews were conducted in English as the respondents were very conversant with the language. One research assistant with postgraduate training in qualitative research transcribed the interviews. To enhance reliability and consistence, the first author reviewed transcripts by listening to all the nine audios and comparing them to the transcripts. Data analysis followed thematic analysis which ‘is a method for identifying, analysing and reporting patterns (themes) within data. It minimally organizes and describes data set in (rich) detail and goes further to interpret various aspects of the research topic’ [34].

The first step in analysing data was the development of codes. Coding process was carried out by two people with the use of NVIVO version 7 (QSR Australia). To ensure quality, the coding process was independently done and any possible differences were discussed to arrive at the final codes. This was followed by the grouping of the codes into categories - groups of content that share a commonality - and these were then developed into broader themes. The process of developing the codes involved interpreting the categories for their underlying meaning, and grouping categories according to patterns [9]. To further promote quality or reliability of the themes, all the authors participated in the process of refining the themes in order to arrive at the final codes. In line with the interview guide, the data analysis process started with mapping the key actors with whom CHAs engage in the community system. This was followed by outlining themes on how CHAs deliver SRH services as well as the ways or conditions through which CHAs negotiate with the elements of the community health system in order to gain acceptability.

### Ethics

The study was approved by to the Excellency in Research Ethics and Science (ERES) Ethics Committee in Zambia before commencing the study. Permissions were sought from the Ministry of Health and relevant district authorities to conduct the study in health facilities. The study was also approved by the National Research Authority. The process of identifying the study participants at the district level started with seeking permission from the District Medical Officers. Informed consent was sought from all eligible participants before they participated in the study. To promote non-coercion, study participants were informed that they were free not participate from the study and that such a decision would not affect their work/ employment status. All interviews were conducted in privacy and strict confidentiality was maintained. To further ensure privacy and confidentiality, we removed all the personal identifiers such as names, age and name of the health facility during the transcription process, writing and dissemination process.

## Results

The results section starts by presenting the key players that CHAs work with in providing their services, and the spaces or settings where the CHAs deliver the services in the community. It then goes on to outline how CHAs negotiate and navigate their way to gain entry and acceptability within the community health system as they deliver SRH services.

### Position of CHAs in the health system

The CHAs were trained for one year and had worked for an average of 6 years in the district at the time of this study. Of the nine CHAs that were interviewed, five were male four were female. Their average age was 32 years. All the CHAs reported performing the roles and responsibilities described in the national strategy.

CHAs worked with several actors in providing SRH in the communities, including nurses and environmental health technicians, community health workers, safe motherhood action groups, and community-based health distributors. Others were the health center committees, neighborhood health committees, teachers and community leaders of various sporting activities, churches as well as traditional authorities (Table 1). The way CHAs positioned themselves in relation to these community actors varied across the district depending on individual relationships and networks established in each community.

**Table 1:** Key players in the community health system

Key players	Number per health post				
	Chipanje Health Post	Chifukizi Health Post	Kalingidi Health Post	Msima Health Post	Mtilizi Health Post
Health center committees	1	1	1	1	1
Neighborhood health committees	6	5	7	6	3
Safe motherhood action members	20	20	21	45	40
Community health workers	3	6	5	3	3
Community-based distributors	4	–		9	–
Environmental health technicians	1	1	1	1	1
Nurses	1	1	1	1	1
Support staff	1	1	1	1	1
Population	5289	5513	5513	4641	3042

Others: Traditional, political, religious and school based leaders, NGOs, Zambia Police

Environmental health technicians are mainly responsible for delivering health sanitation programs in the community. The safe motherhood action groups provide information on various maternal and child health services, encouraging early booking for antenatal services and delivery at health facilities. The community-based health distributors provide different types of family planning methods at household level. In addition to conducting health promotion activities such as sanitation campaigns, the job descriptions of CHAs include the activities performed by the safe motherhood action groups and community-based health distributors. Community leaders and teachers assist CHAs in mobilisation processes.

### Settings or spaces where CHAs deliver SRH education and services

The settings in which the CHAs provided services can be broadly categorized into four groups, namely government managed spaces, family settings, community spaces, and community events (Table 2). They worked with the range of actors outlined in Table 1 to provide a combination preventive, promotive and care services addressing HIV and STIs, family planning, gender-based violence and maternal and child health.

**Table 2:** Summary settings, actors and roles

Spaces or settings	Actors collaborating with CHAs	SRH Roles
Government controlled settings	- Nurses - EHTs - Support staff - SMAGs - NHCs	- delivering babies in some emergency cases though this is not a part of their training and scope of work - promoting and providing family planning - HIV / STI counselling, testing and promoting ART adherence - male circumcision - delivering prenatal and postnatal services - sensitisation against early pregnancy and marriage
Family controlled settings	- CBDs - SMAGs	- promoting early booking for antenatal - promoting and providing family planning - HIV counselling, testing and promoting ART adherence
Community public space	- CHWs - NHCs - Traditional leaders - Councillors and WDC chairperson - Religious leaders	- promoting early booking for antenatal - promoting and providing family planning - promoting HIV/ STI counselling, testing and promoting ART adherence - sensitisation against early pregnancy and marriage
Community events	- Traditional leaders - Captains of netball and football teams - CHWs	- promoting early booking for antenatal - promoting and providing family planning - promoting HIV/ STI counselling, testing and promoting ART adherence - sensitisation against early pregnancy and marriage

### Factors that shape acceptability and integration of CHA-run SRH services

The previous section has documented the settings or spaces through which the CHAs delivered services. This section explores how the CHAs actively negotiated their entry into these spaces to deliver SRH services within the community health system, as well as the struggles that they experienced when doing so.

#### CHA health facility role facilitating the integration process

As indicated, CHAs provided several services at health posts, including treating common illness such as malaria and diarrhea, and delivering babies. Most of the CHAs reported that being at the health post provided a safe space to talk with young people about issues related to SRH problems, integrating the SRH provision into other services. In addition, some CHAs used their health post position to build contacts with young people for further follow up on SRH issues at community level.

*“Since I work here, I make it a point to all young people that come to the health post to talk about sexuality-related health problems such as unwanted pregnancies and STIs, and also the services that are available here”* (CHA 3, male).

#### Know-how and knowledge sharing as a negotiating tool for support from other actors in providing SRH services

Most of the CHAs reported that their one-year training positively facilitated their integration into the community health system. The training gave them the skills needed to provide family planning information and services, enhancing their confidence in relation to other service providers at community level. These actors often viewed CHAs as knowledgeable, who were able to capitalise on this to involve and mobilise them in delivering SRH services among young people.

*“They (community volunteers) know my capacity in terms of training and they always consult us ----- when providing family planning services. They tell us that they are lucky to have us in the community”* (CHA 1, female).

#### Building and bridging relationships

Despite their relative status amongst community health system actors, the CHAs recognised the importance of treating others with respect if they were to draw on support from community-based actors in providing SRH to young people. The CHAs organised regular meetings, which apart from mobilising community actors, facilitated the development of shared purpose among these actors and a positive environment of collaboration and consultation.

*“To be able to work with the community, people look at your behaviour – respect, use of decent language and communication when correcting and approaching others are vital in promoting team work. These attributes are key especially when providing sensitive services to adolescent such as contraception”* (CHA 4, male).

CHAs also promoted acceptability of their role in delivering SRH services among other community actors by acting as brokers. This was done, for example, by CHAs raising the working conditions of other volunteers, such as the lack of consistent payment of incentives by the Ministry of Health during meetings with district officials. This helped CHAs to build relationships in the community health system.

*“When the volunteers do not get an incentive, I talk on their behalf. So they know we have a common agenda, and they will help me with issues in the community, including providing SRH* (CHA 2, male).*”*

#### Embedding SRH into general life skills

Another approach of gaining acceptability for SRH was to integrate this into other health promotion activities such as sanitation and childhood immunization. This strategy was mainly used in the school and church settings and at community events, in order to reduce the sensitivity of the SRH matters, while enabling the CHAs to capture the interest or attention of many people in the community.

*“In schools, you first cool them down, talk about sanitation first, and follow up with assertiveness, while they are still reflecting on those issues you introduce condom and contraception use”* (CHA 6, female).

#### Leveraging on existing social networks

Being born and working in the same community positively facilitated integration of the CHA SRH services into the community and among other community actors. Many CHAs reported that they made use of the trust relationships they had with community volunteers they had previously worked with, before they were engaged as CHAs.

*“My friends (community volunteers) connect me to young people. Since they (adolescents) know them (community volunteers), they do not want to disappoint them (community volunteers) by refusing my services, so I use the opportunity to provide contraception and condoms”* (CHA 1, female).

#### Providing care in the home enhancing social bonds

Acceptability of CHAs in the community health system (among some households) was further facilitated by CHAs’ ability to bring the SRH services as close as possible to the households through household visits, allowing CHAs to become more familiar with and strengthening social bonds with the community. These enhanced social bonds further reinforced the capacity of the community to support and promote SRH rights and services on behalf of the CHAs.

*“Accessing services from the house is what they like most. They like the privacy when we talk about family planning in their households. Because we visit them, they have supported our activities. They complain when we do not visit them often”* (CHA 3, male).

#### Leadership support promoting legitimacy of SRH

The support from the community leaders - including the traditional leaders, head teachers and religious leaders – also facilitated the integration process of the CHA driven services into the community system. This support followed formal introduction of CHAs to the community leaders by the District Health Office, creating legitimacy for their services, and opening up community space for many people to attend CHA-coordinated health promotion activities. Recognition by the leaders, in turn, inspired the CHAs to work even harder, as they felt respected, recognised and trusted.

*“It used to be difficult for us to provide family planning services to adolescents as people did not know us. The problem was further compounded by the religious and cultural barriers with regard to providing contraception to adolescents. Our services have however improved following the support from the traditional leaders”* (CHA 7, female).

### Factors hindering acceptability of CHA SRH services

#### The taboo of discussing sexuality issues

Local cultural norms and values regarding adolescent sexuality and provision of contraception impeded the acceptability of some CHA services. School policy did not favor discussion and distribution of condoms and other contraceptive methods in schools. Largely due to these contextual factors, all the CHAs reported that they emphasised abstinence in schools when teaching the learners, which they reported was in line with the community expectations.

*“For the young ones, we (CHAs) discourage them from engaging in early sexual activities. We fear that the parents may not be happy with us if they know that we encouraged their children to adopt condoms use as a way of preventing pregnancies”* (CHA 6, female).

#### Gender patterns to accessing SRH

Single or unmarried female adolescents were less likely to access the SRH services than males, while boys were reported to be more active participants in school SRH education than girls. Fear of being stigmatized if women sought contraception before marriage is still a barrier to access. Further, some women did not feel free to discuss SRH issues with male CHAs.

*“In terms of sex, it was mainly the boys that accessed the services compared to girls. For females, it was mainly those who are married or had children who utilized the services”* (CHA 7, female).

While a few CHAs supported use of family planning services among girls, others aligned themselves with community social norms, indicating that it was not acceptable for young female adolescents to seek family planning services before they were married.

“*I think that girls who are in school should not use contraception as this may make them addicted to sexual intercourse and affect their performance in class”* (CHA 11, female).

#### Power relations among providers at community level

While most CHAs collaborated well with other actors within the community health system, this was not universally the case. In particular, CHAs sometimes had difficulty in positioning themselves in relation to the neighbourhood health committees, because while NHCs recruited and were supposed to supervise CHAs, in some cases, members of the NHCs were CHWs who had less training than CHAs. Thus when doing community work, the NHC members who were also CHWs, took instruction from and reported to CHAs on activities. However, some NHCs did not want to report to people that they recruited and who they were supposed to supervise. These micro-politics affected the delivery of SRH services as the CHAs depended on the NHCs to mobilise the community.

*“The challenge comes in when you are working with NHCs, I find it difficult to question some of their reports because these people are part of the committee that recruited us, let me just say they are our bosses”* (CHA 8, male).

#### Work schedule affecting social bonds

All the CHAs struggled to fit within their ideal work schedule which required four days of community visits and one day working at their health post. Due to inadequate staff, CHAs often ended up spending more time at the health post, affecting provision of family planning services to people who preferred accessing such services within their homes. CHAs also complained that their failure to engage in community tasks resulted in the community seeing them as members of the formal health system and not as part of the community, thus undermining trust.

*“If I can’t go to the community, then people will consider me as an outsider who cannot be easily be trusted. Such as a situation is problematic given that trust is key to uptake of contraception among the adolescents as there are several myths in the community regarding contraception”* (CHA 1, female).

## Discussion

CHAs successfully worked with different actors to deliver SRH information and reproductive health services at community level. These actors include other government workers, community health workers and community leaders. SRH services were delivered within government-managed spaces, community spaces, family settings, and at specific events. To facilitate acceptability of their SRH services, CHAs used different strategies and occupied different roles and positions depending on the situation, the nature of the context, and the actors with whom they were dealing. CHAs also used a range of strategies to navigate the various elements of community systems. For example, CHAs balanced stressing their professional expertise and linkages with the health facility, while leveraging community connectedness.

The struggles faced by community based health workers (CBHWs) in delivering health interventions in the community health system have been outlined in other studies [9, 17, 22, 23]. It has been noted that as they navigate along this path, CBHWs face different kinds of expectations, and their success in managing these expectations is dependent on the type and quality of support from the formal health system and from social networks within the communities [17]. CBHWs have to struggle to simultaneously balance identities as *“community insider, outsider, and broker”* [17].

Further, effectively navigating with the elements of the community health system in order to foster acceptability of CHW-driven services requires understanding of the relationships and capabilities at the community level. This is important because CHW programs tend to be enabled or constrained by local social norms, power structures [17]. CHAs may themselves share or be unwilling to challenge harmful social norms or promote rights based approaches.

The nature and pattern of the relationships and interactions forged will shape the possibilities for delivery of SRH services in communities [15]. CHAs strengthened community relationships through household visits and community meetings, while drawing on community capabilities such as the community health workers and community leadership, spaces and events to deliver SRH services. The relevance of leveraging on key domains of community capability such as physical and financial assets; capacity for collective action or breadth of participation; and community participation in service delivery and governance has been documented as key to promoting synergies in community health systems [23].

Meanwhile, a range of contextual factors constrained CHAs’ ability to navigate elements of the community health system and promote acceptability of CHA-driven SRH services. One of these issues was gender inequalities and local norms that did not favour access to SRH services by young, unmarried women. General stigmatisation of young people who accessed SRH also affected the acceptability of CHA-driven SRH. Other challenges included limited human resources for health and supplies at the health post, which in turn affected the delivery of SRH services at household level.

Enhancing the acceptability of CHA-driven SRH services requires positively shaping the context to enable CHAs to effectively negotiate with elements of the health systems. There is a need not just to add SRH services to existing services or to extension workers, but also to change the nature of their work to better address adolescent SRH needs [21, 22]. Specifically, addressing contextual elements of the health system would require paying attention to the *cultural contextual issues* such as stigma and negative images of young people related to SRH; the *organisational/network context* such as conflicting roles of CHAs in relation to delivery of SRH services; and the *material-political context* which includes lack of resources to support the delivery of SRH services [35]. Gender analysts further argue for the goal not just to integrate women / girls or men/boys into systems that are problematic, but also to change those services to better address key population needs [22, 23, 36]. Other important aspects that require attention in the context in HIV interventions include consideration of key psycho-social preconditions for participation in sensitive topics such as knowledge, social spaces for critical thinking, a sense of ownership, confidence, and appropriate bridging relationships [37].

### Limitations and strengths

Doing the study in one district only and among the nine CHAs limits generalization of the study findings to other settings. Further, including community members and health workers would have provided valuable perspectives on CHA driven SRH services. The complementary backgrounds and qualifications of the researchers (anthropology, and public health) helped in improving trustworthiness of data and its analysis and interpretation. The rich description of the study context, the phenomena (integration process of CHA- driven SRH services) and providing of the quotations in the text representing a variety of informants enhanced transferability or analytical generalization of the results. Furthermore, the findings of this study informed the design and implementation of a larger study that adopted a mixed methods study design in examining uptake of adolescent SRH services in five provinces and ten districts in Zambia.

## Conclusion

CHAs work with government workers, community health workers and community leaders in delivering SRH services in government-managed spaces, community spaces, family settings and community events. To facilitate acceptability of their SRH services, CHAs play a variety of roles such as mobilisers, advisors, supervisors and brokers. CHAs also use several strategies in facilitating the acceptability of their SRH services at community level. These strategies include using their health facility services to build supportive relations for SRH services, negotiating acceptability through sharing knowledge with other volunteers, soliciting the involvement of volunteers in delivering SRH through holding regular joint meetings, and acting as brokers between the community health workers, safe motherhood action groups, community-based distributors and the Ministry of Health. The CHAs also used their existing social networks to deliver SRH services to adolescents while also reducing the sensitivity of some SRH topics by embedding them into general life skills. Collective action towards addressing SRH issues was promoted through building social bonds with the community through undertaking household visits and engaging with community leaders.

Meanwhile socio-contextual issues such as the taboo of discussing sexuality issues, inadequate human resources, and insufficient supplies at the health post as well as gender norms limited the acceptability of some CHA SRH services. Helping CHAs navigate these challenges will require developing innovative strategies that will ensure increased technical, material and social support from both formal and community health systems actors in delivering sexual and reproductive health services.

## Data Availability

The datasets during and/or analysed during the current study are available from the corresponding author on reasonable request.

## Abbreviations

HCs: Health centres
CHAs: Community Health Assistants
CHW: Community health worker
HPs: Health posts
LMICs: Low and middle income countries
NHCs: Neighbourhood Health Committees
SRH: Sexual and reproductive health
